# Replicating Current Procedural Terminology code assignment of rhinology operative notes using machine learning

**DOI:** 10.1002/wjo2.188

**Published:** 2024-05-28

**Authors:** Christopher P. Cheng, Ryan Sicard, Dragan Vujovic, Vikram Vasan, Chris Choi, David K. Lerner, Alfred‐Marc Iloreta

**Affiliations:** ^1^ Department of Medical Education Icahn School of Medicine at Mount Sinai New York New York USA; ^2^ Department of Otolaryngology Icahn School of Medicine at Mount Sinai New York New York USA; ^3^ Department of Otolaryngology University of Miami Miller School of Medicine Miami Florida USA

**Keywords:** CPT code, machine learning, natural language processing, rhinology, skull base

## Abstract

**Objectives:**

Documentation and billing are important and time‐consuming parts of an otolaryngologist's work. Given advancements in machine learning (ML), we evaluated the ability of ML algorithms to use operative notes to classify rhinology procedures by Current Procedural Terminology (CPT®) code. We aimed to assess the potential for ML to replicate rhinologists' completion of their administrative tasks.

**Study Design:**

Retrospective cohort study.

**Setting:**

Urban tertiary hospital.

**Methods:**

A total of 594 operative notes from rhinological procedures across six CPT codes performed from 3/2017 to 4/2022 were collected from 22 otolaryngologists. Text was preprocessed and then vectorized using CountVectorizer (CV), term frequency‐inverse document frequency, and Word2Vec. The Decision Tree, Support Vector Machine, Logistic Regression and Naïve Bayes (NB) algorithms were used to train and test models on operative notes. Model‐classified CPT codes were compared to codes assigned by operating surgeons. Model performance was evaluated by area under the receiver operating characteristic curve (ROC‐AUC), precision, recall, and F1‐score.

**Results:**

Performance varied across vectorizers and ML algorithms. Across all performance metrics, CV and NB was most overall the best combination of vectorizer and ML algorithm across CPT codes and produced the single best AUC, 0.984.

**Conclusions:**

In otolaryngology applications, the performance of basic ML algorithms varies depending on the context in which they are used. All algorithms demonstrated their ability to classify CPT codes well as well as the potential for using ML to replicate rhinologists' completion of their administrative tasks.

## Key points

- Models were trained and tested to classify 549 operative notes into six CPT codes in under 1 s. Performance varied by algorithm and CPT code. Overall, CV vectorization with logistic regression and Naive Bayes algorithms most often performed best.
- Using machine learning to generate rhinology CPT codes can replicate rhinologists' manual CPT assignments. The performance of these basic ML models establishes a basis of comparison for use in evaluating more advanced ML models' replication of physician administrative tasks.

## INTRODUCTION

Each patient's electronic health record (EHR) is an integral part of their interaction with the healthcare system and each EHR contains a comprehensive amount of information about each patient.^1^, ^2^ Part of this documentation consists of a massive corpus of free text that helps document patients' history and treatment course to justify billing.^1^ Billing in the United States is facilitated by Current Procedural Terminology (CPT®) codes maintained by the American Medical Association; each procedure has a unique code.^1^, ^3^, ^4^ In coding, accuracy is important as miscoding can result in mistaken billing and affect hospital and provider revenue.^5^ Some providers further argue that the individual act of parsing and identifying appropriate billing codes contributes to greater administrative workload for each physician.^6^, ^7^ That administrative workload can be alleviated by a tool that can replicate physicians' ability to assign CPT codes to their operations.

With the advent of machine learning (ML) techniques such as natural language processing (NLP) that allow for quantifiable analysis of free text, the wealth of information in patient documentation can now be leveraged for quantitative applications.^8^, ^9^, ^10^ NLP has been previously used in the medical setting to emphasize important report findings, summarize a document's themes, predict ICD‐O morphology codes, and predict CPT codes from diagnostic text.^11^, ^12^, ^13^, ^14^, ^15^, ^16^ The most recent procedures for extracting CPT codes from diagnostic text have used advanced deep learning models that performed well in other fields such as pathology and orthopedics, although these studies only included a limited range of basic algorithms. Moreover, ChatGPT is a tool with potential uses for written healthcare data. Given the continued emergence of novel technologies that can analyze text in healthcare, it is necessary to establish a basis for comparison for novel techniques using currently existing technologies.

To the authors' knowledge, no study has been performed on classifying intra‐operative notes by CPT code in the field of rhinology. Therefore, the purpose of this study is to assess the performance of basic ML and NLP techniques at extracting CPT codes from intraoperative surgical notes at a large, urban academic medical center to establish an educational and proof‐of‐concept foundation for future research and application of ML techniques. By validating the ability of ML tools to replicate surgeons' assignments of CPT codes, these findings can help elaborate on the feasibility of automating a notable administrative task for rhinologists.

## MATERIAL AND METHODS

### Study design and compliance

This retrospective cohort study developed and assessed different combinations of word vectorization techniques and machine language algorithms applied for the purpose of analyzing select rhinological operative notes to predict the relevant CPT codes. This study was approved by the Mount Sinai Institutional Review Board under expedited review and granted a waiver of informed consent and HIPAA authorization.

### Inclusion criteria

Data was retrieved via the Mount Sinai Data Warehouse. The inclusion criteria for data used to train and test models in this study instructed the Data Warehouse to gather all available CPT codes for rhinological procedures performed at Mount Sinai Hospital from March 1, 2017 to April 30, 2022. Up to 100 cases available per CPT code were selected for use in this analysis. The internal definitions and operative note count for each CPT code evaluated are detailed in Table 1. Twenty‐two unique surgeons wrote the notes collected using both templates and original writing and reviewed the codes assigned to their operations. The single exclusion criterion applied excluded CPT codes with fewer than 20 operative notes available from the aforementioned time period, as such a small sample size would not be sufficiently robust for training ML models that would offer meaningful insight into the viability of their application. Ultimately, this resulted in the retrieval of the operative notes of eight CPT codes (30520, 31255, 31267, 31276, 31288, and 61580, see Table 1) assigned to rhinological procedures.

**Table 1:** All rhinological CPT codes collected according to the inclusion criteria of this study.

CPT	Definition	Entries
30520	Septoplasty or submucous resection, with or without cartilage scoring, contouring or replacement with graft	100
31255	Surgical endoscopy of nose and sinus with complete ethmoidectomy	100
31267	Surgical endoscopy of nose and sinus with maxillary antrostomy and removal of tissue from maxillary sinus	100
31276	Surgical endoscopy of nose and sinus with exploration of frontal sinus and removal of tissue from frontal sinus	100
31288	Surgical endoscopy of nose and sinus with sphenoidotomy and removal of tissue from sphenoid sinus	89
61580	Craniofacial approach to extradural lesion of anterior cranial fossa, with lateral rhinotomy, ethmoidectomy, and sphenoidectomy	37

### Word preprocessing and vectorization techniques

At the simplest level, words need to be converted into numbers to be leveraged for use in model training and prediction. Different preprocessing and vectorization techniques translate bodies of text in different ways that may contribute to variations in the efficacy between individual ML models applied to unique and individualized use cases.

First, words needed to be preprocessed to be simplified and standardized across different grammatical representations such as tense, punctuation and capitalization. Using regular expressions, words were converted to lowercase while new line whitespace, punctuation and numerical digits were removed. Stop words, the commonly used words in a given language, were removed (e.g., “the,” “is,” “and”) using the stopword function of the Python Natural Language Toolkit (NLTK). NLTK's WordNetLemmatizer was used to reduce words to their base form, known as a lemma; for example, “inserted” and “inserts” would be reduced to their common lemma: “insert.”^17^

After preprocessing, vectorization was carried out with three of the most traditional and established vectorization techniques in NLP: CountVectorizer (CV), Term Frequency‐Inverse Document Frequency (TFIDF), and Word2Vec (W2V). The former two were used according to their scikit‐learn 0.23.2 implementation, while the latter relied on the Gensim 4.1.2 implementation. All three vectorizers were run by their default parameters.^18^, ^19^, ^20^

CV converts a body of text into a matrix of numbers that are assigned based on the frequency with which each word appears in the body of text.

TFIDF assigns a numerical matrix similarly to CV but also considers the frequency with which a given word appears in every other body of text it preprocesses, reducing the weight of certain words that appear commonly across all bodies of text.

Finally, W2V is a vectorizer built on neural networks that create numerical representations, or “word embeddings,” of words that are unique to and reflect the context in which they're found. In practice, W2V is meant to produce word embeddings whose mutual similarities reflect the semantic and syntactic similarities of the texts from which they were produced.

### ML algorithms

Given the novelty of applying ML to otolaryngology and in particular to the subspecialty of rhinology, this study aimed to establish a baseline understanding of its performance capabilities. Therefore, the ML algorithms trained in this study were five of the most traditional and tested algorithms: Bagging Decision Tree, Random Forest Decision Tree, Support Vector Machine (SVM), Logistic Regression and Naïve Bayes (NB), and were used according to their implementations in scikit‐learn 0.23.2.

#### Decision tree (DT)

The DT algorithm classifies entries according to its tree of “decision nodes” developed according to the training data it is given. Using the training data, which is a set of data entries and their corresponding correct classification, the nodes are arranged into branches where each decision node leads to another and so on until ultimately a classification is made. The algorithm can then be used to classify new data entries. Two of the most notable DT implementations were tested for this study: bagging and random forest (RF).^21^

##### Bagging

Default parameters used for the bagging DT algorithms as implemented by scikit‐learn 0.23.2's BaggingClassifier, which reduces variance and overfitting in classification output. With bagging, the decision made by each node takes into consideration *all* the features available in the data set (in this study's case, the specific word vectors).^22^

##### RF

RF is similarly intended to reduce variance and overfitting for a DT algorithm but instead of each node being trained to make decisions according to all features, it selects random subsets of features to make decisions. In theory, the DTs formed are more independent of one another.^23^

#### SVM

SVMs find a hyperplane boundary to distinguish between two classes and classify inputs accordingly; it does so using a statistical approach that calculates dot products between data points that form the graphical border of a given classification. The dot products are synthesized to create a margin line that distinguishes between two classes. For this data set, given that vectorized operative notes are not in a two‐dimensional space, a kernel trick was used that “flattens” the multidimensional vectors of the data set into a higher dimensional space that is linearly separable.^24^

#### Logistic regression

As with SVM, a logistic regression defines a boundary to distinguish between two classes and classify inputs accordingly. However, in contrast, it does so by calculating the probability of a given input belonging to a given class using the sigmoid function. From these probability‐generated classifications, the algorithm then generates a hyperplane boundary that best separates classifications and thus classifies subsequent inputs accordingly.^25^

#### NB

The NB algorithm uses Bayes' theorem of probability to assume that all features of the data set it is given are independent of one another; although this assumption may not be true in many cases, it still facilitates efficient classification. These probabilities, based on the values of the assumed‐to‐be “independent” features for a given input, then guide the algorithm to make classifications.^26^

### Evaluation criteria

The models were evaluated using the following methods:

#### Receiver operating characteristics (ROC)‐area under the curve (AUC)

A ROC curve is a probability curve that plots false positive rate against true positive rate; the AUC is a measure of how well the model distinguishes between two classes, or in this study how well each model is able to correctly classify a note by the CPT code for which it was actually billed.^27^

#### Precision

The precision of a classification algorithm, otherwise known as the positive predictive value, represents the probability in this study that the classification of an operative note as a certain CPT code was in actuality billed by a surgeon as such.^28^

#### Recall

The recall of a classification algorithm, otherwise known as sensitivity, represents the probability that an operative note billed by a surgeon as a given CPT code will actually be classified by the algorithm as such.^28^

#### F1‐score

F1‐score measures accuracy and is defined as the harmonic mean of precision and recall, calculated by the following formula: 2×(precision×recall)/(precision+recall).^28^

## RESULTS

In total, 549 operative notes fulfilled the inclusion criteria of this study across six different CPT codes. Collectively, the time taken to classify a given CPT code for up to 100 operative notes was less than 1 s. The performance of all combinations of word vectorization techniques and ML algorithms is illustrated in Tables 2, 3, 4, 5 and summarized as follows.

**Table 2:** Area under the curve characteristics for all CPT codes using different word vectorization techniques and machine learning algorithms.

		AUC		
		CV	TFIDF	W2V
30520	DT	0.758	0.716	0.739
	RF	0.713	0.690	0.722
	SVM	0.731	0.746	0.775
	LR	0.720	0.759	0.776
	NB	0.734	0.709	0.709
31255	DT	0.594	0.559	0.547
	RF	0.593	0.578	0.535
	SVM	0.577	0.447	0.640
	LR	0.618	0.643	0.704
	NB	0.701	0.558	0.558
31267	DT	0.532	0.502	0.441
	RF	0.491	0.472	0.473
	SVM	0.515	0.422	0.520
	LR	0.445	0.583	0.681
	NB	0.650	0.513	0.513
31276	DT	0.481	0.537	0.473
	RF	0.507	0.511	0.495
	SVM	0.515	0.530	0.496
	LR	0.451	0.588	0.643
	NB	0.671	0.577	0.577
31288	DT	0.706	0.709	0.663
	RF	0.698	0.706	0.708
	SVM	0.754	0.717	0.719
	LR	0.742	0.733	0.765
	NB	0.789	0.719	0.719
61580	DT	0.971	0.977	0.732
	RF	0.977	0.974	0.961
	SVM	0.867	0.933	0.967
	LR	0.981	0.982	0.983
	NB	0.984	0.981	0.981

Abbreviations: CV, CountVectorizer; DT, Decision Tree; NB, Naive Bayes; RF, Random Forest; SVM, Support Vector Machine; TFIDF, Term Frequency‐Inverse Document Frequency; W2V, Word2Vec.

**Table 3:** Precision values for all CPT codes using different word vectorization techniques and machine learning algorithms.

		Precision		
		CV	TFIDF	W2V
30520	DT	0.852	0.828	0.822
	RF	0.811	0.820	0.830
	SVM	0.833	0.861	0.901
	LR	0.818	0.826	0.864
	NB	0.795	0.753	0.753
31255	DT	0.671	0.667	0.645
	RF	0.646	0.681	0.646
	SVM	0.676	0.650	0.650
	LR	0.671	0.716	0.649
	NB	0.766	0.650	0.650
31267	DT	0.643	0.626	0.609
	RF	0.610	0.633	0.630
	SVM	0.633	0.621	0.621
	LR	0.633	0.619	0.621
	NB	0.722	0.621	0.621
31276	DT	0.642	0.653	0.655
	RF	0.634	0.649	0.661
	SVM	0.661	0.650	0.650
	LR	0.692	0.644	0.648
	NB	0.765	0.650	0.650
31288	DT	0.766	0.766	0.766
	RF	0.766	0.766	0.766
	SVM	0.780	0.793	0.741
	LR	0.780	0.769	0.739
	NB	0.883	0.741	0.741
61580	DT	0.946	0.946	0.949
	RF	0.942	0.942	0.942
	SVM	0.942	0.946	0.946
	LR	0.942	0.946	0.955
	NB	0.981	0.958	0.958

Abbreviations: CV, CountVectorizer; DT, Decision Tree; NB, Naive Bayes; RF, Random Forest; SVM, Support Vector Machine; TFIDF, Term Frequency‐Inverse Document Frequency; W2V, Word2Vec.

**Table 4:** Recall of the positive class (or sensitivity) values for all CPT codes using different word vectorization techniques and machine learning algorithms.

		Recall of the positive class		
		CV	TFIDF	W2V
30520	DT	0.861	0.842	0.836
	RF	0.830	0.836	0.842
	SVM	0.842	0.867	0.897
	LR	0.824	0.842	0.873
	NB	0.764	0.806	0.806
31255	DT	0.770	0.764	0.776
	RF	0.782	0.782	0.782
	SVM	0.776	0.806	0.806
	LR	0.770	0.800	0.800
	NB	0.594	0.806	0.806
31267	DT	0.721	0.715	0.721
	RF	0.727	0.733	0.727
	SVM	0.733	0.788	0.788
	LR	0.733	0.776	0.788
	NB	0.685	0.788	0.788
31276	DT	0.691	0.691	0.697
	RF	0.715	0.715	0.715
	SVM	0.715	0.806	0.806
	LR	0.721	0.770	0.794
	NB	0.721	0.806	0.806
31288	DT	0.812	0.812	0.812
	RF	0.812	0.812	0.812
	SVM	0.818	0.848	0.861
	LR	0.818	0.818	0.848
	NB	0.679	0.861	0.861
61580	DT	0.952	0.952	0.958
	RF	0.945	0.945	0.945
	SVM	0.945	0.952	0.952
	LR	0.945	0.952	0.958
	NB	0.970	0.964	0.964

Abbreviations: CV, CountVectorizer; DT, Decision Tree; NB, Naive Bayes; RF, Random Forest; SVM, Support Vector Machine; TFIDF, Term Frequency‐Inverse Document Frequency; W2V, Word2Vec.

**Table 5:** F1‐scores for all CPT codes using different word vectorization techniques and machine learning algorithms.

		F1‐score		
		CV	TFIDF	W2V
30520	DT	0.854	0.831	0.826
	RF	0.814	0.823	0.833
	SVM	0.836	0.848	0.884
	LR	0.821	0.824	0.862
	NB	0.776	0.740	0.740
31255	DT	0.710	0.707	0.704
	RF	0.707	0.717	0.707
	SVM	0.714	0.720	0.720
	LR	0.710	0.727	0.716
	NB	0.636	0.720	0.720
31267	DT	0.675	0.665	0.660
	RF	0.663	0.675	0.672
	SVM	0.675	0.694	0.694
	LR	0.675	0.688	0.694
	NB	0.700	0.694	0.694
31276	DT	0.665	0.671	0.674
	RF	0.672	0.679	0.685
	SVM	0.685	0.720	0.720
	LR	0.705	0.701	0.713
	NB	0.738	0.720	0.720
31288	DT	0.786	0.786	0.786
	RF	0.786	0.786	0.786
	SVM	0.796	0.808	0.796
	LR	0.796	0.790	0.790
	NB	0.728	0.796	0.796
61580	DT	0.948	0.948	0.948
	RF	0.944	0.944	0.944
	SVM	0.944	0.948	0.948
	LR	0.944	0.948	0.956
	NB	0.973	0.958	0.958

Abbreviations: CV, CountVectorizer; DT, Decision Tree; NB, Naive Bayes; RF, Random Forest; SVM, Support Vector Machine; TFIDF, Term Frequency‐Inverse Document Frequency; W2V, Word2Vec.

Overall, AUC values ranged from 0.441 to 0.984. For three CPT codes (30520, 31255, and 31267) the best vectorizer and algorithm combination was Word2Vec and logistic regression, whereas for the other three codes (31276, 31288, and 61580) it was CV and NB. On average across all CPT codes, the highest‐performing vectorizer was CV with an average AUC of 0.682, whereas the highest‐performing ML algorithm was logistic regression with an average AUC of 0.711. Ironically, the combination of CV and logistic regression yielded the lowest AUC across all combinations involving at least one of the two. All AUC results can be seen in Table 2.

Overall, precision values ranged from 0.609 to 0.981. For five CPT codes (31255, 31267, 31276, 31288, and 61580) the best vectorizer and algorithm combination was CV and NB; for the lone exception (30520), the best combination was Word2Vec and SVM. On average across all CPT codes, the highest‐performing vectorizer was CV with an average precision of 0.763, whereas the highest‐performing ML algorithm was NB with an average precision of 0.759. Accordingly, the combination of CV and NB yielded the highest precision across all combinations: 0.819. All precision values can be seen in Table 3.

Overall, recall values of the positive class (sensitivity) ranged from 0.594 to 0.970. For four CPT codes (31255, 31267, 31276, 31288) the best vectorizer and algorithm combination was tied four ways between the different permutations of TFIDF and Word2Vec with SVM and NB (the one exception being TFIDF with SVM on code 31288, which was worse than the other three). For 30520, the best combination was Word2Vec with SVM alone; for 61580, it was CV with NB. On average across all CPT codes, the highest‐performing vectorizer was Word2Vec with an average sensitivity of 0.827, whereas the highest‐performing ML algorithm was SVM with an average sensitivity of 0.834. Accordingly, the combination of Word2Vec and SVM yielded the highest sensitivity across all combinations: 0.852. All sensitivity values can be seen in Table 4.

Overall, F1‐scores ranged from 0.636 to 0.973. For three CPT codes (31267, 31276, 61580) the best vectorizer and algorithm combination was CV and NB, whereas there were different superior combinations for the other four codes. For 30520, the best combination was Word2Vec with SVM; for 31255, TFIDF with logistic regression; and for 31288, TFIDF with SVM. On average across all CPT codes, the highest‐performing vectorizer was Word2Vec with an average F1‐score of 0.778, whereas the highest‐performing ML algorithm was SVM. Accordingly, the combination of Word2Vec and SVM yielded the highest F1‐score across all combinations: 0.794. All F1‐scores can be seen in Table 5.

Overall, recall values of the negative class (specificity) ranged from 0.571 to 1.00. For four CPT codes (31255, 31267, 31276, and 31288) the best vectorizer and algorithm combination was tied four ways between the different permutations of TFIDF and Word2Vec with SVM and NB (the one exception being TFIDF with SVM on code 31288, which was worse than the other three). For 30520, the best combinations were Word2Vec with SVM and Logistic Regression; for 61580, they were TFIDF and Word2Vec with NB. On average across all CPT codes, the highest‐performing vectorizer was Word2Vec with an average specificity of 0.969, whereas the highest‐performing ML algorithm was SVM with an average specificity of 0.972. Accordingly, the combination of Word2Vec and SVM yielded the highest specificity across all combinations: 0.997, a result shared with the combination of Word2Vec and NB. All specificity values can be seen in Table 6.

**Table 6:** Recall of the negative class (or specificity) values for all CPT codes using different word vectorization techniques and machine learning algorithms.

		Recall of the negative class		
		CV	TFIDF	W2V
30520	DT	0.940	0.940	0.918
	RF	0.940	0.940	0.940
	SVM	0.925	0.977	0.992
	LR	0.902	0.955	0.992
	NB	0.812	0.984	0.985
31255	DT	0.947	0.940	0.940
	RF	0.970	0.962	0.970
	SVM	0.955	1.00	1.00
	LR	0.947	0.985	0.992
	NB	0.571	1.00	1.00
31267	DT	0.900	0.900	0.915
	RF	0.923	0.923	0.931
	SVM	0.923	1.00	1.00
	LR	0.923	0.985	1.00
	NB	0.754	1.00	1.00
31276	DT	0.850	0.842	0.872
	RF	0.887	0.880	0.865
	SVM	0.872	1.00	1.00
	LR	0.857	0.955	0.985
	NB	0.774	1.00	1.00
31288	DT	0.930	0.930	0.930
	RF	0.930	0.930	0.930
	SVM	0.930	0.972	1.00
	LR	0.930	0.937	0.993
	NB	0.641	1.00	1.00
61580	DT	0.981	0.981	0.981
	RF	0.975	0.975	0.975
	SVM	0.975	0.981	0.987
	LR	0.975	0.981	0.981
	NB	0.968	0.994	0.994

Abbreviations: CV, CountVectorizer; DT, Decision Tree; NB, Naive Bayes; RF, Random Forest; SVM, Support Vector Machine; TFIDF, Term Frequency‐Inverse Document Frequency; W2V, Word2Vec

## DISCUSSION

The goal of this study was to establish a foundation for using ML algorithms to replicate surgeons’ assignments CPT codes to their rhinological operative notes. Doing so can help automate physician administrative tasks. A total of 18 combinations formed from three vectorization techniques and six ML algorithms were used to train the classification of six CPT codes from 549 unstandardized surgeons’ notes written by 22 unique surgeons, which lends adequate generalizability to this pilot study.

Performance varied across vectorizers, ML algorithms, and evaluation metrics used. Across all performance metrics, CV and NB was most commonly the best combination of vectorizer and ML algorithm for assigning a given CPT code (3/6 for ROC‐AUC, 5/6 for precision, 1/6 for sensitivity, 0/6 for specificity and 3/6 for F1‐score). This finding suggests using CV with NB may be a good starting point for developing a viable ML model to assign CPT codes to a given set of rhinological operative notes before trying other combinations in the pursuit of optimal performance according to ROC‐AUC and F1‐score. However, if one is pursuing optimal sensitivity and specificity, results show that combining Word2Vec with SVM may be a better option. Interestingly among all evaluation metrics, the individual best‐performing vectorization and ML algorithms do not necessarily produce the best performing vectorization/ML algorithm combinations. These diverse results suggest that using basic ML algorithms requires a significant degree of tailoring to the specific CPT code being assessed and the evaluation metric used to assess it. This study did not find any given vectorizer or model to be “one‐size‐fits‐all.”

Compared to Levy et al.'s study investigating CPT code classification of pathology reports using 93,039 reports written by more than 20 pathologists across five CPT codes, which achieved an average ROC‐AUC of 0.554 for a SVM model, the model in this study scored an average ROC‐AUC of 0.659.^1^ Kim et al.'s study investigating CPT code classification of 391 spine surgery operative notes written by one surgeon across 15 CPT codes achieved an average ROC‐AUC of 0.94 for its RF model, as compared with the average ROC‐AUC of 0.656 in this study.^16^ Possible reasons for the difference from Levy et al.'s results include that pathology report documentation may have greater variation in words used as compared with rhinology, which may affect basic ML algorithms' ability to accurately classify CPT codes. Possible reasons for the difference from Kim et al.'s results include that internal consistency of operative note formatting (i.e. only classifying notes written by a single surgeon) offers greater performance benefits than attempting to classify multiple surgeons' notes.

Notably, the value of this study's objective to establish a baseline performance level for basic ML algorithms is reinforced by the difference in the performance of “basic” ML algorithms in this study as compared to those of Levy et al. and Kim et al. For example, although both aforementioned studies selected a single basic algorithm (SVM for Levy et al., RF for Kim et al.) for classification performance comparison against algorithmic augmentations such as XGBoost or more advanced algorithms such as BERT and the Long Short Term Memory Network, this study's results have shown the significant variation among results depending on the model, vectorizer and CPT code in question. Indeed, NB and logistic regression models scored higher on average than the SVM and RF models in this study on the same ROC‐AUC evaluation metric used by Levy and Kim. This finding has implications for future work in applying ML in healthcare and the selection of baseline ML algorithms against which the performance of more advanced algorithms can be compared.

Furthermore, from a practical perspective the findings of this study reiterate both the feasibility and potential benefits of using ML in a clinical setting that have been proposed in similar studies, such as the automation of identification of CPT codes from free text, the scale at which this process is carried out and the reduction in manpower that this process would afford.^29^

The limitations of this study include the following: the notes were taken from a single urban hospital, and further work can be done to assess cross‐institution performance. Another limitation is that the study considered only six rhinological CPT codes. A larger and more diverse data set can facilitate improvements in assessing the accuracy and generalizability of ML models. Finally, all algorithms were used with their default parameters. All this suggests that further optimizations in the model are possible.

More advanced deep learning algorithms such as the Bidirectional Encoder Representations from Transformers (BERT) algorithm are a promising avenue for development of a classification model for CPT codes with consistently high performance. As its developers Devlin et al. suggest, BERT can “reduce the need for many heavily‐engineered task‐specific architectures.”^30^ Further, integrations with ChatGPT offer another promising avenue for assistance with administrative tasks, although it may independently assign CPT codes and not necessarily replicate rhinologists' classification patterns as seen in this study.

## CONCLUSION

This study demonstrated the feasibility of using a wide range of basic ML algorithms to automatically replicate rhinologists' classification of rhinological operative notes by CPT code. Indeed, when compared with past studies in other specialties such as pathology and orthopedics, this study has demonstrated the value of assessing and evaluating a broader range of basic ML algorithms given the variability in their performance depending on CPT code. Though performances varied across the algorithms/algorithm combinations, evaluation criteria, and CPT codes, high percentages were achieved in a number of cases that allude to the promise of applying ML methods for replicating rhinologists' performance of administrative tasks, especially with future application of more advanced ML algorithms and tools.

## AUTHOR CONTRIBUTIONS

All authors have contributed to the conceptualization, drafting, analysis and revision of the manuscript and approved its contents.

## CONFLICT OF INTEREST STATEMENT

The authors declare no conflict of interest.

## ETHICS STATEMENT

This study was approved by the Mount Sinai Institutional Review Board under expedited review and granted a waiver of informed consent and HIPAA authorization.

## Data Availability

The corresponding author should be contacted regarding interest in the raw data or model parameters of this study.
